# Sensing Movement: Microsensors for Body Motion Measurement

**DOI:** 10.3390/s110100638

**Published:** 2011-01-10

**Authors:** Hansong Zeng, Yi Zhao

**Affiliations:** Laboratory for Biomedical Microsystems, Department of Biomedical Engineering, The Ohio State University, Columbus, OH 43210, USA

**Keywords:** motion sensors, human vestibular system, accelerometer, gyroscope, liquid-state motion sensor, artificial hair cell *motion* sensor, thermal convection accelerometer

## Abstract

Recognition of body posture and motion is an important physiological function that can keep the body in balance. Man-made motion sensors have also been widely applied for a broad array of biomedical applications including diagnosis of balance disorders and evaluation of energy expenditure. This paper reviews the state-of-the-art sensing components utilized for body motion measurement. The anatomy and working principles of a natural body motion sensor, the human vestibular system, are first described. Various man-made inertial sensors are then elaborated based on their distinctive sensing mechanisms. In particular, both the conventional solid-state motion sensors and the emerging non solid-state motion sensors are depicted. With their lower cost and increased intelligence, man-made motion sensors are expected to play an increasingly important role in biomedical systems for basic research as well as clinical diagnostics.

## 1. Introduction

Motion sensing is a critical sensing modality that plays an important role in medical practice. For instance, head rotation and body orientation are the input signals for human balance prosthesis [1,2]; the movement of chest wall needs to be precisely monitored when a ventilation machine is used to support human breath [3,4]; the body motion characteristics also need to be evaluated during the rehabilitation process of disabled people [5–7]. The current clinical solution for motion sensing is to use a camera based motion capture system [8,9], where the body motion is derived from the movement of multiple feature points attached on the body. Although effective, this technique is obtrusive and expensive. It is also difficult to be integrated into a modern medical system, such as portable medical device and point-of-care (POC) medication. Recently, microscale motion sensing technologies have gained dramatic advances, which have significantly propelled the development of human balance prosthesis [10–13], sports medicine [14–16], radiotherapy [17–19], and biomechanical research [20–22]. In particular, rapid development of micro-electro-mechanical-systems (MEMS) with high accuracy, high reliability and multiple functionalities has provided a powerful tool set for body motion sensing [23,24]. Over the past two decades, research on microscale motion sensors has received extensive attention, and continues to be an active domain.

**Figure 1.**
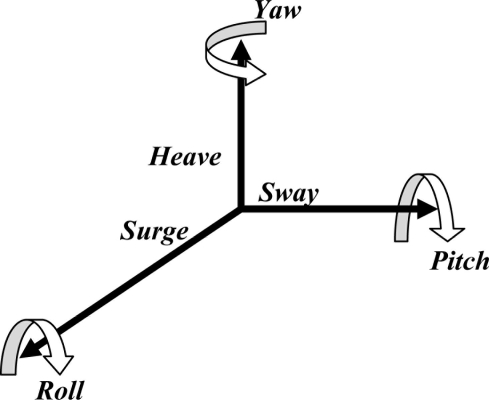
The six independent variables fully describing the motion characteristics of an object.

Generally speaking, the motion characteristics of an object, such as a human subject, an organ, or a tissue (e.g., solid tumor), can be described by six independent variables. As schematized in Figure 1, sway, heave and surge are linear motions along the three perpendicular coordinate axes in the space; roll, pitch and yaw are rotational movements with respect to the three perpendicular directions [25]. In order to accurately measure the motion characteristics of an object, a sensing system with six degree-of-freedom (DOF) sensing capability is required.

The human vestibular system possesses a simple but delicate structure that can simultaneously and accurately detect the six independent variables, which are subsequently interpreted by the central and peripheral neural systems to keep body balance and maintain gaze stability. Among the engineering modalities, linear motion and rotational motion are detected by accelerometers and gyroscopes, respectively [26–30]. Cantilever based accelerometers have attracted tremendous interest during the past decades, and are widely available for motion sensing. Each accelerometer includes a mass-spring-damper system where the linear acceleration can be derived from the displacement of the proof mass. The rotational motion is measured by micromachined gyroscopes, which can be categorized mainly in two groups, linear vibratory gyroscope and torsional gyroscopes [31,32]. Basically, a micro-gyroscope is composed of two sets of mass-spring-damper systems, one for driving and the other for sensing. Besides the solid-state sensors, many innovative microscale technologies that are not based on solid state materials also provide promising solutions for body motion sensing. In particular, microsensors based on liquid-state proof mass gain particular attentions because their sensing principles are closer to those of the natural motion sensors. These sensors fulfill the low frequency requirement that is critical for human body motion measurement.

In this paper, state-of-the-art microscale motion sensing technologies that can be utilized for human body motion determination will be introduced. The review starts with the introduction of a natural motion sensing system, namely the human vestibular organ. Its anatomy and physiological function are elaborated. This is followed by a comprehensive review of conventional solid-state sensors. The representative configurations, design principles, key fabrication approaches and sensing mechanisms of micromachined accelerometers and gyroscopes are described. Afterwards, several innovative motion sensing technologies based on non solid-state materials are introduced, including the biologically inspired hair cell accelerometer, liquid droplet based motion sensors, and thermal convection based accelerometers. Finally, representative applications of man-made sensors for body motion measurement are elaborated. The development and expansion of this field in the near future are also discussed.

## 2. Human Vestibular System

**Figure 2.**
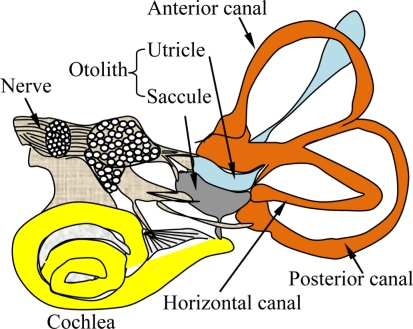
Schematic of the human vestibular system showing the three perpendicular semi-canals and the otolith (utricle and saccule) in the inner ear.

Posture recognition and motion sensation in living creatures is accomplished by the coordination of a number of different organs. For example, humans can identify their motion states using eyes, ears, the vestibular system, joints, skin, along with many other inner organs. The subject can adjust his gesture accordingly in real time using the feedback mechanism coordinated by the central and peripheral neural systems. Among these organs, human vestibular system is a naturally established motion sensing apparatus locating in the inner ear [33]. It senses the body acceleration and head rotation, which are subsequently transferred to the central and peripheral neural systems for balance control and gaze stabilization. The anatomy of a vestibular system is illustrated in Figure 2 [34].

**Figure 3.**
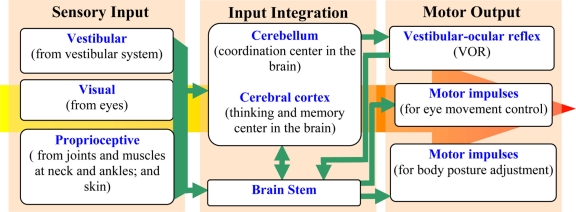
Balance control in human body.

The system is composed of two sets of end organs, namely the semicircular canals and the otoliths. The semicircular canals are in charge of rotational movement sensing, while the otoliths are in charge of translational movement sensing. The semicircular canals are filled with a body fluid, named endolymph, which moves relatively to the canal wall when the subject experiences head rotation. In response to the fluid movement, the hair cell bundles on the canal wall bend and generate pulses of action potentials. The frequency of the bioelectric pulses is proportional to the intensity of the hair cell bending and reflects the angular rates of the head rotation. In order to perceive the movement in the three-dimensional space, the vestibular system in each ear contains three semicircular canals perpendicular to each other, which are called horizontal, anterior and posterior semicircular canals respectively. These canals are arranged in a way that each canal in one ear has a parallel counterpart in the other ear. With such a configuration, when a canal in one ear is stimulated, the corresponding counterpart in the other ear is inhibited. This push-pull fashion allows human beings to differentiate the direction of the rotational movements. The semicircular canals system is an indispensable part of the vestibule-ocular reflex (VOR), which stabilizes images on the retina during head movement. The VOR receives the head rotation information and produces eye movement in the opposite direction to preserve the image in the center of the visual field. Figure 3 illustrates the relationship between the sensory inputs and the motors output, showing the role of VOR in the adjustment of eye movement. If the VOR system is impaired, blurred vision occurs, even under small head tremors, leading to vertigo and dizziness. Moreover, appropriate motor impulses for postural adjustments cannot be achieved, which results in symptoms that accompany body unsteadiness.

While the semicircular canals are responsible for angular rate measurement, the otoliths sense the linear acceleration. The otolith in each ear contains two small organs called utricle and saccule. Because of their physical orientations in the head, the utricle is sensitive to the motion in the horizontal direction, and the saccule senses the movement in the vertical direction. Similar to the semicircular canals, the otoliths are also composed of endolymph and hair cells. Upon an external motion, the inertial movement of endolymph stimulates the hair cells by bending them. The bioelectric signals generated by the hair cells are transferred to and interpreted by the brain. Along with the inputs from the eyes and the joints, the brain obtains the balance states of the human body and sends commands to the motor system for postural control. Interestingly, although the gravity is equivalent to a linear acceleration along the vertical direction from a kinetic view, the vestibular system can distinguish the gravity from the linear acceleration quite well by not fully understood mechanisms.

Since the vestibular system is an indispensable part of the human balance system, its disorder causes a range of symptoms including blurred vision, vertigo, chronic dizziness, and increased fall risk. According to the clinical survey, more than 6.2 million adults in the United States have reported chronic balance problems and more than 1 billion U.S. dollars are spent each year on the medical care of such diseases [35]. Vestibular disorder can be caused by various reasons, such as injury, infection, neural diseases, surgery, drug poisoning, etc., making pharmaceutical treatments alone ineffective for their treatment [36]. It is clear that vestibular prosthesis is necessary to help patients with balance problems. Nowadays, many man-made motion sensors with the functions comparable to that of the vestibular system have been developed. In the following sections, state-of-the-art technologies of man-made motion sensors that have potentials for evaluation of body motion disorder or vestibular prosthesis are reviewed.

## 3. Solid-State Motion Sensors

### 3.1. Cantilever Based Accelerometers

The use of cantilever based accelerometers for assessing human body movement was first proposed in the 1950s, although the devices in the early stage were somewhat unreliable, large and expensive [37]. Thanks to the revolutionary advancement of microfabrication technologies, state-of-the-art micro-accelerometers have become more accurate, reliable, smaller and cost effective [38–42]. Their applications in biomedical areas have been extensively explored, significantly changing this field of body motion measurement [43–45].

**Figure 4.**
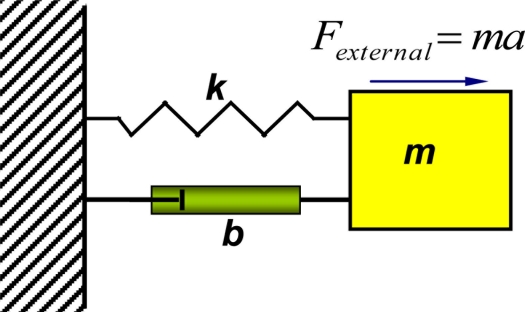
A second-order mass-spring-damper system representing a cantilever based accelerometer.

A typical cantilever based accelerometer structure can be represented by a mass-spring-damper system, as illustrated in Figure 4. The key component is a proof mass suspended by a compliant beam (cantilever) anchored to a supporting frame. The inertial force generates a relative movement between the proof mass and the supporting frame, and induces mechanical stress within the cantilever. Both of the relative movement and the mechanical stress can indicate the external acceleration.

Equation (1) is the second-order mathematical model that describes the movement of the proof mass as a function of the applied external acceleration [46]:

(1) $$m\frac{d^{2}x}{{\mathrm{dt}}^{2}}+b\frac{\mathrm{dx}}{\mathrm{dt}}+\mathrm{kx}-m\times a\;\text{sin}\;\omega t=0$$

where *m* is the proof mass, *x* is the displacement of the proof mass, *t* is the time variable, *b* is the damping coefficient, *k* is the spring constant of the cantilever, *a* is the external acceleration to be determined, and ω is the angular frequency of the external acceleration. In a cantilever based microaccelerometer, the displacement of the proof mass is often on the order of a few μm or less. Such a small displacement needs to be converted to a measurable physical signal to determine the external acceleration. Many sensing mechanisms have been utilized to determine the cantilever displacement and hereby the acceleration, through capacitive, piezoresistive, piezoelectric or tunneling current measurement.

#### 3.1.1. Capacitive Accelerometer

**Figure 5.**
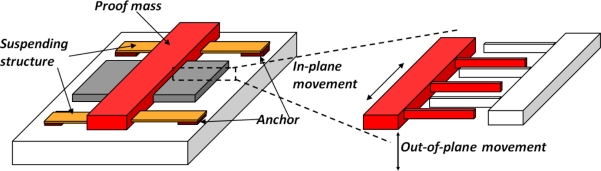
Schematic of the capacitive measurement of the proof mass movement.

Capacitive accelerometers are one of the most widely used solid-state motion sensors on the market [47,48]. The relative displacement between the proof mass and the supporting frame is derived from the change of electrical capacitance when the movable electrode plate displaces either in-plane or out-of-plane with the stationary supporting frame (Figure 5). In practice, the movable electrodes and the stationary frame are often in the form of interdigitated electrode fingers (a.k.a. comb-drive structures). The overlapping area of the movable fingers and the stationary fingers and the gap distance between the fingers are important parameters determining the measuring sensitivity [49].

#### 3.1.2. Piezoresistive and Piezoelectric Accelerometers

Micromachined piezoresistive accelerometers were first described by Roylance and Angell [50], and are currently used in various industrial applications. The configuration of the cantilever structures in piezoresistive accelerometers is similar to those in capacitive accelerometers [51], while their electrical measuring mechanisms are different. In piezoresistive accelerometers, a piezoresistor is often patterned on a thin suspending cantilever which connects the proof mass and the supporting frame. Due to the mechanical flexibility of the cantilever, a large mechanical strain occurs as the external acceleration displaces the proof mass. The strain is derived from the electrical resistance change in the piezoresistor.

Piezoresistive accelerometers can be fabricated by both surface micromachining and bulk micromachining [52–54]. By using a piezoresistor as the sensing component, this type of accelerometers is advantageous due to the relatively simple configuration and fabrication. Nonetheless, piezoresistive accelerometers are highly vulnerable to the temperature variation. Improved designs includes the use of a large proof mass, integration with a temperature compensation circuitry, and the monolithic implementation with CMOS electronics [55,56].

Piezoelectric accelerometers have the similar configuration with the piezoresistive counterparts, but measuring the acceleration from the electrical voltage induced by the mechanical displacement of the cantilever [57]. A notable difference is that piezoelectric accelerometers only respond to dynamic signals while the piezoresistive sensors can measure displacements under low and zero frequencies.

#### 3.1.3. Tunneling Accelerometers

The tunnel effect describes the phenomenon that occurs when a conductive sharp tip and a counter electrode are positioned at a small gap distance on the order of 10 Å and set at a DC voltage bias, established an electric tunneling current between the tip and the electrode before the two parts contact each other [58]. The electric tunneling current changes exponentially with the gap distance. Such exponential relationship provides an ultrasensitive approach for displacement detection, and has been utilized for the implementation of a micromachined tunneling accelerometer with high resolution [59]. In a typical configuration, the proof mass is attached to the conductive tip and positioned at a small gap to the counter electrode. During the measurement, the tunneling voltage remains constant. The proof mass is brought close to or away from the counter electrode upon an external acceleration. The change of tunneling current reflects the displacement of the cantilever and is a measure of the external acceleration. It is reported that the tunneling current changes by a factor of two for each Å of displacement, providing an ultra high sensitivity. In addition, since the tunneling tip is only effective within the local area around the tip (1 μm^2^), the device can be further miniaturized without affecting the electrical measurement. The tunneling tip is usually fabricated by KOH etching of (100) single crystal silicon followed by metal deposition. Anodic bonding is usually employed to assemble the tip and counter electrode [60].

### 3.2. Gyroscopes

Gyroscopes refer to the sensors that measure the rotary rate of an object. A micromachined gyroscope utilizes the Coriolis effect to convert the rotary motion of the subject into a measurable linear motion. The rotary rate can therefore be determined using the above described sensing mechanisms of measuring linear accelerations.

**Figure 6.**
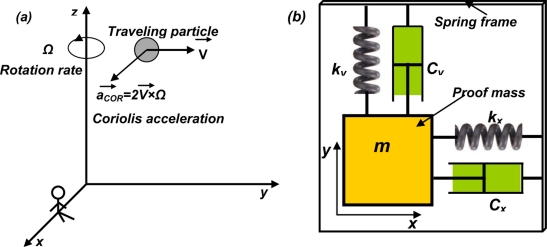
Schematic of the vibratory gyroscope. **(a)** illustration of Coriolis effect; and **(b)** the mass-spring-dasher system of a vibratory gyroscope.

As known, Coriolis effect refers to the generation of an imaginary force (Coriolis force) perpendicular to the moving direction of the subject within a rotating coordinate system [61]. This is illustrated in Figure 6(a) and briefed described as below. Imagine that an observer sitting on the *x*-axis of a rotating coordinate system around z-axis observes a moving particle traveling at the velocity *V* in the space. Because of the rotating coordinate system, the particle has a trajectory toward the *x*-axis with an acceleration *2V × Ω* from the view of the observer. This apparent acceleration observed in a rotating coordinate system is called Coriolis acceleration, proportional to the rotation rate of the coordinate system (*Ω*) and the traveling velocity of the object (*V*). According to this phenomenon, a micromachined gyroscope without any rotary parts can be designed, which consists of two sets of mass-spring-damper systems positioned in the perpendicular directions [Figure 6(b)]. One set is in the driving mode and the other is in the sensing mode. Each of the mass-spring-damper sets is basically a linear accelerometer. During the operation, the proof mass in the driving set is actuated at a certain frequency. Upon a rotary movement of the frame, the Coriolis acceleration generated in the orthogonal direction provides inputs to the sensing set, which is measured by the sensing mode accelerometer.

The governing equations of the system in Figure 6(b) can be described by two mass-spring-damper systems coupled in two orthogonal directions [61]:

(2) $$\begin{array}{l} m\ddot{x}+c_{x}\dot{x}+k_{x}x=\tau_{x}\\ m\ddot{y}+c_{y}\dot{y}+k_{y}y=\tau_{y}-2m\Omega_{z}\dot{x} \end{array}$$

where *x* and *y* are the displacements of the proof mass in *x* and *y* directions respectively, *c_x_* and *c_y_* are the damping coefficients in the two directions, *k_x_* and *k_y_* are the spring constants in the two directions, *τ_x_* and *τ_y_* are the external force in the driving and sensing modes, and Ω*_z_* is the angular rate of the coordinate system to be determined. It should be pointed out that the above configuration only presents one typical form of linear vibratory micromachined gyroscope. Many other configurations, including but not limited to linear vibratory gyroscopes [62] and torsional gyroscopes [63], are also available.

### 3.3. Packaging and Integration

Packaging and integration are essential for solid-state micromachined inertial sensors. Packaging not only provides a mechanical housing to avoid the damping, manage the thermal transfer, isolate the mechanical shock and protect the sensors from a harsh chemical environment, but also electrically connects the sensor to the outside environment and isolates it from external electromagnetic interference. The packaging also reduces the variation of damping conditions among devices designed and fabricated through the same process [64]. The packaging techniques should be considered at the design phase since different packaging methods may require different fabrication processes and configurations. In practice, anodic bonding, eutectic bonding, thermal fusion bonding or glass frit bonding techniques are regularly used [46].

The mechanical sensing components in solid-state inertial sensors need to be integrated with the controlling and measuring circuitry. Two types of integration methods, namely hybrid integration and monolithic integration, are usually used [55]. Hybrid integration employs wire bonding to electrically connect the sensing component with its controlling IC, where the electrical interconnects are exposed outside the packaging. Monolithic integration, on the other hand, fabricates the sensor and its controlling IC simultaneously within a single step. It thus minimizes the contact issues and parasitic effects, which is good for many CMOS-compatible fabrication processes [46]. Detailed discussion of packaging and integration is beyond the scope of this review, and can be found in literatures [46,51–55,64].

## 4. Non Solid-State Motion Sensors

From the above description of solid-state motion sensors, it is not difficult to figure out that motion detection using man-made solid-state sensors is somewhat different from that of natural motion sensing systems which relies on non solid-state materials. One notable difference is the range of measuring frequency. The resonance frequency of a miniaturized motion sensor using solid-state proof mass is often on the order of a few kHz or above [61], while the typical frequency of body motion is often below 10 Hz [3,6,7,10]. To reduce the resonance frequency, a slender cantilever (*i.e.*, large length to width ratio) is often needed. This however increases fabrication and packaging complexity, and reduces the mechanical robustness of the sensors. Inspired by natural motion sensors, non solid-state motion sensors have been recently proposed as another man-made motion sensing mechanism, which utilize various different sensing principles for body motion detection. Specifically, sensors using fluidic or gaseous materials as the proof mass are depicted. Their distinct characteristics are compared with solid-state sensors in terms of frequency response, fabrication, packaging and integration methods. Since most of these non solid-state motion sensors are emerging during the past two years, there have not been extensive reports that apply these sensors for body motion measurement. This section therefore focuses mainly on the working principles and sensor developments.

### 4.1. Hair Cell Based Motion Sensor

**Figure 7.**
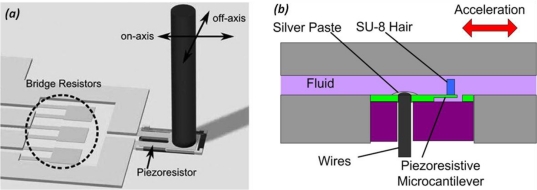
Artificial hair cell sensor. **(a)** the schematic view; and **(b)** the cross sectional schematic of a prototype.

As above mentioned, the human vestibular system measures body motion based on the bending of hair cell bundles. Inspired by this, an artificial hair cell sensor using polymeric and metallic materials was developed [65]. The utility of such a sensor in motion measurement is also demonstrated [66]. Figure 7(a) shows the schematic of the artificial hair cell, where a cantilever with a piezoresistive strain-gauge is suspended in the atmosphere and an SU-8 pillar is attached on the distal end of the cantilever. An on-axis force towards the SU-8 pillar induces a mechanical strain at the cantilever base and is measured by a piezoresistor. The artificial hair cell sensor is integrated within a fluidic system to mimic the natural vestibular system [Figure 7(b)]. By placing the artificial hair cell sensor inside a channel filled with water, the device possesses the geometry similar to that of a semicircular canal in the vestibular system. When an external acceleration is applied, the fluid inside the channel has a relative movement with the sensor and bends the hair cell to a certain extent corresponding to the acceleration magnitude. Different from a cantilever based accelerometer, the system uses the liquid fluid as the proof mass, while the cantilever sensor is a measuring apparatus rather than a sensing component. The sensor shows a good linearity and a sensitivity of 16.9 *mV/g* with a water-filled channel system under accelerations at 225 Hz.

### 4.2. Liquid Droplet Based Accelerometer

**Figure 8.**
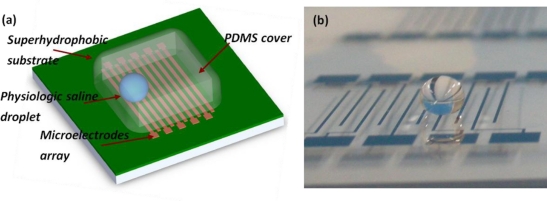
Liquid droplet based accelerometer. **(a)** the schematic view; and **(b)** a prototype without the PDMS cover.

Another instance of motion sensor utilizing the liquid as the proof mass is the liquid droplet based motion sensor, which uses a liquid droplet as the proof mass. The basic structure and configuration of such a sensor can be found in [67] and is seen in Figure 8.

It consists of a hydrophobic substrate patterned with an array of microelectrodes and a 20 μL saline droplet resides on top of the substrate. When an external acceleration is applied, the ionic droplet moves relatively with the substrate surface due to the inertial effect. This relative motion is captured by the electrode array as the conductive ionic droplet changes the electrical impedance between every two adjacent microelectrodes. In the droplet based motion sensor, the wettability of the substrate surface determines the dynamic contact angle hysteresis and surface friction of the droplet movement. A superhydrophobic surface is desired to obtain a sensor with low measuring threshold, high sensitivity and good linearity. The governing equation of a liquid droplet based sensor can be expressed as [67]:

(3) $$m\frac{d^{2}x}{{\mathrm{dt}}^{2}}+b\frac{\mathrm{dx}}{\mathrm{dt}}-\left(m\times a-F_{\mathrm{threshold}}\right)=0$$

where *m* is droplet mass, *x* is the relative displacement, *t* is the time variable, *b* is the damping coefficient, *a* is the magnitude of the external acceleration, and *F_threshold_* denotes the capillary force due to the dynamic contact angle hysteresis.

**Figure 9.**
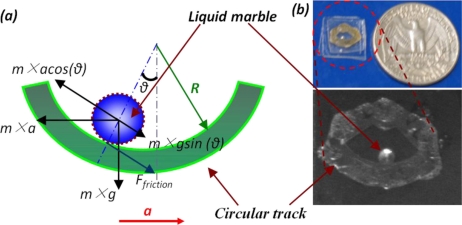
The improved design of liquid-state motion sensor with a curved channel. **(a)** the schematic and **(b)** the prototype.

It is noted that comparing to the governing equation of a solid-state accelerometer, Equation (3) lacks a term representing the spring. As the result, the liquid droplet does not return to the original point, which makes it incapable of continuous measurement. An improved design is presented by utilizing a curved channel instead of a planar substrate to hold the liquid droplet [68], shown in Figure 9. As the liquid droplet displaces, the tangential component of the gravity along the channel surface provides the restoring force and moves the droplet back to the origin. The governing equation in this situation can be expressed as [68]:

(4) $$R^{2}\ddot{\theta }+\xi R\dot{\theta }+g\;\text{sin}(\theta )=a_{0}\;\text{sin}(2\pi \cdot f\cdot t)\;\text{cos}(\theta )$$

where: *R* is the radius of the circular channel,

$\xi =\frac{b}{m}$ is the dimensionless damping coefficient and
$\theta =\frac{x}{R}$ is the angular displacement of the droplet,
*g* is gravity,
*a_0_* is the magnitude of the external acceleration and
*f* is the frequency of the external acceleration.

**Figure 10.**
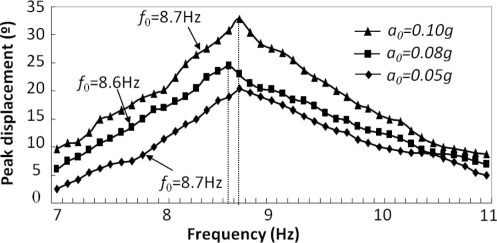
The frequency response of a liquid droplet based motion sensor, where the resonance frequency is around 8.7 Hz.

A non-linear spring is involved that allows continuous measurement. The relationship between the droplet displacement and the frequency and magnitude of external acceleration shows the sensor has the greatest response around 8.7 Hz (Figure 10), which is within the frequency range of human body motion (<10 Hz). It is therefore feasible to apply liquid droplet based motion sensor for low frequency body motion sensing.

### 4.3. Liquid-Metal Based Accelerometer

**Figure 11.**
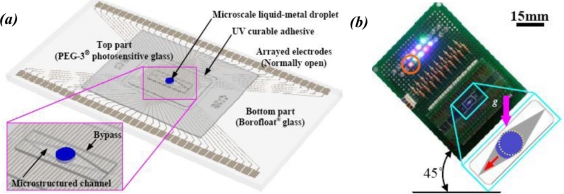
Liquid-metal droplet based accelerometer. **(a)** the schematic view; and **(b)** a prototype tilted at 45°.

A similar approach is to measure the external acceleration based on the motion of a microscale liquid-metal (LM) droplet [69]. The concept is shown in Figure 11(a). The LM droplet initially stays in the center of the glass channel, connecting the electrodes across the middle of the channel. When an external acceleration is applied, the droplet will move towards one end of the channel and be deformed by the channel. As the droplet moves, it covers the corresponding electrodes. The position of the LM droplet, and hereby the external acceleration, can thus be indicated by the light-emitting diodes (LED) connected to the electrodes [Figure 11(b)]. Linear accelerations as large as 40 *g* can be measured.

### 4.4. Thermal Convection Based Accelerometer

**Figure 12.**
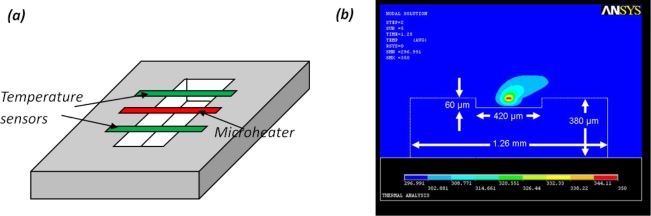
Thermal convection based accelerometer. **(a)** the typical configuration; and **(b)** an external acceleration induces temperature field asymmetry around the microheater.

Thermal convection based accelerometer uses air as the inertial component [70]. A suspending electrical microheater is used to heat the surrounding air [Figure 12(a)]. Once a thermal equilibrium is developed, the temperature field has a radial pattern where the air close to the heater is at a higher temperature, and the air at a distance is at a lower temperature. At a steady state, the temperature distribution is symmetric. When an external acceleration is applied, the temperature field around the heater changes due to the inertial effect of the air, resulting in an asymmetric temperature field, which can be predicted using finite element analysis [Figure 12(b)] [71]. Such an asymmetric temperature field around the heater is measured by the temperature transducers patterned around the heaters, which can be either thermocouples or thermistors.

Since the suspending heating element increases the fabrication complexity and is less robust to vibration shocks, an improved design is to pattern the heaters on a surface with low thermal conductivity. For example, porous silicon has been used as the thermal isolation material between the microheater and the substrate [71].

Like sensors using liquid materials as the proof mass, thermal convection-based accelerometers also have good sensing performance within the frequency range below 100 Hz, which covers the frequency range of body motion. Representative characteristics of solid-state accelerometers and non solid-state motion sensors are compared in Table 1.

It is seen that although all the systems are based on inertial effects, they possess vastly different characteristics. The unique characteristic of each sensing type renders it more suitable for some applications over others. For biomedical applications, sensors with biocompatible materials, suitable measurement ranges and relatively low resonant frequencies are particularly desirable.

## 5. Applications of Motion Detection

Current motion detection and measurement technologies include the use of camera recording systems and solid-state motion sensors. In particular, camera based recording systems based on tracking of multiple feature points of the subject are widely used in biomedical, military, entertainment, sports and robotics applications. Since such imaging based motion tracking is not the focus of this review, interested readers can refer to the literature [72,73] for more details. Here, we confine our discussion within the use of solid-state portable microsensors, which has long existence and mature fabrication, packaging and integration technologies. We hope the discussion of engrafting solid-state sensors into biological researches and clinical practices could shed light on the use of non solid-state motion sensors in the future.

### 5.1. Fall Evaluation

As mentioned at the beginning of this review, one of the most straightforward applications of motion sensors is to evaluate balance disorders. A common occurrence in the aging population is unexpected falls. Based on the clinical survey, one out of every three 65-year and elder falls each year. This often causes severe problems such as hip fractures and even deaths. In the clinical practice, Berg Balance Scale (BBS) and the Timed Up and Go test (TUG) are the standard methods for evaluation of balance capacity. In these behavioral tests, the patient is asked to perform a series of motion tasks, e.g., sitting unsupported, reaching forwards while standing, and turning around while carrying a full cup of water. The time used for accomplishing such tasks is the measure of the patient’s balance capability [74,75]. These methods require a doctor’s input and tend to be subjective. A quantitative alternative solution of balance assessment is proposed using a commercial tri-axial accelerometer (ActivPal^−^ Trio) [76], where the torso acceleration of both healthy and balance disordered patients under different clinical conditions are measured. The comparison with BBS and TUG testings shows that the accelerometry data inversely correlates with BBS scores and positively correlates with TUG values with statistical significance. The difference of the accelerometry data between the fallers and the non-fallers can clearly sort different balance conditions, which shows the feasibility of using solid-state accelerometers for quantitative balance evaluation. Many research groups and industrial partners are developing algorithms that judge the existence of an emergency fall [77,78], where the characteristics of the acceleration change during the fall are investigated to give an early warning to the subject for gestural adjustment. The development of the algorithms and the design of the sensor network are beyond the scope of this review, and can be found in the literature [79–81].

### 5.2. Balance Prosthesis

The balance prosthesis system usually involves a vibrotactile feedback system that contains a motion sensing system and a tactor actuation system. The sensing system uses solid-state accelerometers and gyroscopes to detect the body orientation and provides this information to the patient through the mechanical actuators. As a result, the patient can adjust his motor system for controlling body posture. A representative vibrotactile feedback system with three capacitive accelerometers can be found in [82], which effectively reduces the sway in patients with bilateral vestibular loss. A motion sensor with six DOFs sensing capacity applied in a vibrotactile system can lead to the improvement of dynamic gait index (DGI, a fall risk factor) in community older adults by observing significant improvement of mediolateral sway control in the subjects [83]. With the current development of solid-state motion sensors, a sensor with six DOFs sensing capacity can be implemented within a chip whose planar area is as small as 5 mm × 5 mm, similar to that of human vestibular organ, which make the implantable balance prosthesis possible. However, despite of the extensive studies about implantable artificial vestibular system [84,85], clinical demonstration of vestibular prosthesis has not been available. This is largely due to the lack of understanding of the interface between the vestibular system and the neural system. In addition, the biocompatibility and long-term reliability of the solid-state sensors, and the signal processing for appropriate data interpretation still need investigation.

### 5.3. Sport Medicine

In sports medicine, motion sensors can be used to measure the intensity of the physical activities, and hereby the energy expenditure during the exercise. For example, the acceleration and the oxygen consumption during treadmill walking and stair walking are successfully correlated in a recent study [86]. The exercise activities of patients with chronic heart failure are monitored using pedometers, a conventional scale inertial sensor with the similar working principle as the solid-state accelerometer but only counts the steps that a subject has walked [87]. Moreover, utilizing a network of solid-state sensors, different activity types, posture and gaits can be recognized, which could provide a comprehensive measure of energy expenditure [88]. When combined with other types of sensors, e.g., thermal sensors for body temperature monitoring and pressure sensor for blood pressure monitoring, these sensors are expected to provide a better estimate of energy expenditure and metabolic work, which contribute greatly to rehabilitation.

### 5.4. Remote Patient Surveillance

Personalized healthcare service, especially monitoring of daily activities is passionately advocated by U.S. healthcare practitioners. Giant healthcare and electronic companies including Intel, Qualcomm, Philips, General Electric, *etc.* are working aggressively in this emerging area [89]. People with suddenly reduced physical activities need special and/or immediate attention, even if the reduction is not recognized by the subjects themselves. For example, deterioration of chronic diseases such as chronic heart failure, diabetes, and Alzheimer’s disease usually correlates with decreased activities [90]. Wearable motion sensors carried by the patient can transfer information about the reduced physical activities in a timely fashion through a wireless sensor network to help the clinician in charge reach a treatment decision. Remote patient surveillance is also critical for the hospitals, where miniaturized motion sensors can be integrated on the hospital beds as well as on the patient body. Combining the measurement obtained from other types of sensors (e.g., pressure sensors), these motion sensors give the physical activities of the patient to decide whether he needs critical and emergent care [e.g., the patients in Intensive Care Unit (ICU)]. This practice is expected to reduce considerably the cost of critical care.

### 5.5. Improving Radiation Oncology

Position monitoring of tumors during radiation therapy is particularly essential for an effective cancer treatment. Even a nearly imperceptible movement may have a substantial negative impact on a patient’s outcome [91]. The position change of a solid tumor can be caused by two events, the mobility of the tumor organ itself and the movement of the patient’s body, e.g., breathing. In order to properly deliver the radiation dose to a defined region, vigilant and active monitoring of the tumor position is required. Solid-state accelerometers are extensively used for this purpose. Depending on the two sources of movement, both tumor position monitoring (organ-level) and body motion monitoring (body-level) can be implemented. Bandala reported a tumor motion tracking method using wireless inertial sensors [25], which contains a three-axis accelerometer, a single-axis gyroscope, a dual-axis gyroscope, a Bluetooth module for wireless data transmission and a microcontroller equipped with the tracking algorithm. The miniaturized navigation system tracks the tumor motion comprehensively with six DOFs. The system has shown satisfactory functionality when tested *ex vivo*. Researchers also monitored body motion to indirectly measure the tumor position, which does not require implanting the sensor into the tumor organ and simplifies the medical implementation. For example, a low-cost wireless accelerometer is placed on patient’s skin to detect the head motion, which predicts the brain tumor position during the radiation. These studies demonstrate the feasibility of improving radiation oncology efficacy using miniaturized motion sensors [92].

## 6. Outlooks and Concluding Remarks

As aforementioned, in contrast to the solid-state sensors that have been widely used for body motion measurement, the applications of non solid-state motion sensors are still in the initial stage. The implementation of body motion sensing using liquid-state sensors or air based sensors is yet to come. Nonetheless, sensors using non solid-state proof masses possess unique characteristics as outlined below, which may make themselves the next paradigms of motion sensors for biomedical applications.

Different from solid-state sensors whose resonance frequency is often on the order of kHz or higher, non solid-state sensors have a much lower resonance frequency, which are often on the order or lower than a couple of hundred Hz. For example, the primary resonance of a hair cell sensor is at 225 Hz and the secondary resonance at 115 Hz. The liquid droplet sensor can have a resonance frequency below 10 Hz. The thermal convectional sensor works best under 100 Hz. Given that the frequency of body motion is usually within the low frequency domain on the order of a few Hz or below, these non solid-state sensors have large response and are adequate for body motion detection. In addition, non solid-state sensors often have simple configurations and are less vulnerable to mechanical shocks. By eliminating the suspending cantilever structures, the mechanical robustness of these sensors can also be improved. Although it is too early to envision a clear market potential for non solid-state motion sensors, the intelligence of these sensors adds new insights to the knowledge base. They are expected to play an increasingly important role in body motion sensing in the future.

The continuous market growth of body motion sensors is governed by both cost and intelligence. Packaging and integration of solid-state sensors, which account for a large fraction of the sensor cost, will remain as a critical cost determinant in the future. Instead of using a single sensor, the use of sensor networks for comprehensive body motion detection has gained and will continue to gain extraordinary attention. The integration and coordination of multiple sensors for appropriate interpretation of specific body activities are essential for the success of intelligent sensors. In addition, the human-sensor interface will be increasingly important, especially for the implantable sensors. With the rapid development of personalized patient care and the technological advances of portable motion sensors that are integrated within the consuming electronic products, e.g., iPhone^−^, the emerging field of portable personalized medical care products, which are supported by open source intelligence worldwide, are expected to grow. This is not only essential for the solid-state motion sensors, but also applicable for non solid-state sensors if they could be engrafted on such products.

In summary, a comprehensive review of state-of-the-art technologies of motion sensors for body motion detection is presented. The anatomy and physiological function of the natural human motion sensor system are introduced, followed by the elaboration of typical configurations, and sensing principles and key fabrication approaches of conventional solid-state motion sensors and the emerging non solid-state motion sensors. The applications of these sensors for body motion measurement are introduced, and the future development of the field is commented. Although not all-inclusive, this review aims to cover the critical conventional sensing modalities as well as typical emerging technologies which may play a leading role in the market of body motion sensing in the near future.

## Figures and Tables

**Table 1.** Comparison of typical man-made inertial sensing systems.

	**Solid-state accelerometers** [41,42,61]	**Artificial hair-cell accelerometer** [66]	**Droplet-based sensors** [67–69]

**Proof mass material**	Solid state materials (e.g., polycrystalline silicon) ±50 g (airbag)	Water	Physiological saline Mercury
**Measurement range**	±2 g (Vehicle stability system) ±1 g (Navigation)	Not available	±40 g [69]
**Sensitivity**	1–2 mV/g(Piezoresistive) 20 fF/g	16.9 mV/g at 225 Hz	114.35 °/g at 10 Hz
**Resonant frequency**	1 k–100 kHz	225 Hz	2–10 Hz
**Packaging and Integration**	Vacuum Package	No vacuum package required	No vacuum package required
**Power consumption**	0.4 mW	Not available	90 μW
